# Vitamin D, muscle strength and function in South Asian women aged ≥ 60 years living in the North of England: a cross-sectional observational study

**DOI:** 10.1007/s00394-025-03787-7

**Published:** 2025-10-09

**Authors:** Sabeen Zahra, Fraser Wiggins, Bernard M. Corfe, Elizabeth A. Williams

**Affiliations:** 1 Healthy Lifespan Institute and the Division of Clinical Medicine, School of Medicine and Population Health, The University of Sheffield, Beech Hill Road, Sheffield, S10 2RX UK; 2 Present Address: Faculty of Medical Sciences, Human Nutrition and Exercise Research Centre, Population Health Sciences Institute, Newcastle University, Newcastle Upon Tyne, NE2 4HH UK; 3 York Trials Unit, University of York, York, YO10 5DD UK

**Keywords:** Vitamin D, Older adults, South Asian, Skeletal muscle strength, Function

## Abstract

**Purpose:**

The importance of vitamin D is well established for bone health and there is some evidence that inadequate/deficient vitamin D status is associated with reduced skeletal muscle strength and physical function in older adults. Most of this evidence for the muscular effect has come from white population groups and the evidence base is sparse for other ethnic groups. This study investigates the relationship between vitamin D status, muscle strength and function in UK South Asian women aged ≥ 60 years.

**Methods:**

This cross-sectional study included 120 community-dwelling Indian and Pakistani women, aged ≥ 60 years living in the North of England. Circulating blood 25(OH)D concentration was assessed by HPLC–MS using finger prick blood samples; functional capacity was assessed using handgrip strength, single and repeated chair stands, timed up and go, and balance test. Regression analysis was used to analyse the relationships between vitamin D status and muscle strength and function.

**Results:**

The median (IQR) age of the women was 66 (64–73) years. Forty-seven percent of the women were vitamin D inadequate/deficient. Around forty-one percent of the women reported taking a daily vitamin D supplement, 86% of whom were vitamin D sufficient (≥ 50 nmol/L). In non-vitamin D supplement users 32% were vitamin D sufficient. Women with vitamin D sufficiency had significantly better single chair stand (*p* = 0.045), repeated chair stand (*p* = 0.01), and balance (*p* = 0.040) test than those with deficiency/inadequacy. No difference was observed in handgrip strength and timed up and go.

**Conclusion:**

In this group of South Asian women aged ≥ 60 years, vitamin D deficiency/inadequacy was common in those not taking vitamin D supplements. Inadequate/deficient vitamin D status was associated with poor performance of lower limb muscle function, but no association was found with handgrip strength and timed up and go. The associations between supplementation, vitamin D status and lower limb strength helps support a rationale for targeted supplementation in this population of older UK South Asian women.

**Supplementary Information:**

The online version contains supplementary material available at 10.1007/s00394-025-03787-7.

## Introduction

Vitamin D is a fat-soluble steroid pro-hormone that plays a critical role in calcium homeostasis and bone health. Vitamin D is also thought to have a mechanism of action in the maintenance of skeletal muscle mass, strength and function through genomic (regulation of DNA transcription) and non-genomic (activation of signalling pathways) pathways mediated through involvement of vitamin D and the vitamin D receptor in skeletal muscle tissue [1]. There is also evidence that low vitamin D status is associated with impaired skeletal muscle mass, strength, and function, and an increased risk of sarcopenia [2]. The principle sources of vitamin D are dermal Ultraviolet B (UVB) photo-conversion of 7-dehydrocholesterol, dietary vitamin D and dietary supplements. UVB induced vitamin D synthesis in the skin is influenced by a number of factors including skin tone/pigmentation (since melanin acts as a barrier to UVB rays) [3], age-related decline in the concentration of 7-dehydrocholesterol [4], duration of sunlight exposure and skin area exposed [5], season of the year [6] and latitude [7]. As a result people of South Asian descent living in the UK are particularly susceptible to vitamin D deficiency due to skin type and low cutaneous sunlight exposure, coupled with a low dietary vitamin D intake [8–10].

Vitamin D intake and status in the UK is a public health concern. The UK reference nutrient intake (RNI) for vitamin D for adults and children over the age of 4 years is 10 µg/day (from all sources) [11]. Supplements make an important contribution to achieving the RNI as illustrated through the UK National Diet and Nutrition Survey (NDNS). The NDNS reported the mean (SD) daily vitamin D intake from dietary sources in women aged 65 years and over was 2.8 (1.9) µg/d and the daily mean vitamin D intake from all sources (including dietary supplements) was reported to be 8.3 (14.3) µg/d [12]. Data on intake and supplement use in South Asian groups living in the UK is limited with the best available evidence coming from Darling et al. [8] who used the UK biobank to quantify vitamin D intake from food and and considered supplement use in 8024 UK South Asians (Bangladeshi, Indians and Pakistani) aged 40–69 years. The daily median vitamin D intake through diet for South Asian women in the cohort was 1 µg/d (IQR 1.6) (similar in men) and vitamin D supplement use in women just 39% (23% in men); with people of Indian ethnicity more likely to use a supplement containing vitamin D than those of Bangladeshi and Pakistani ethnicity [8]. Further analysis of the UK Biobank using a sample of 2927 South Asian women (3506 South Asian men) revealed 89% of the South Asian women (Indian, Pakistani and Bangladeshi) had vitamin D insufficiency ( < 50 nmol/L) and 50% had severe deficiency ( < 25 nmol/L) (94% and 58% respectively in South Asian men) [13]. In comparison 53.7% of white UK biobank participants (n = 422,907) were found to have vitamin D insufficiency (≤ 50 nmol/L) [14] and the NDNS has reported just 13% of women aged ≥ 65 years had a vitamin D status < 25 nmol/L [12].

A positive relationship between vitamin D status and skeletal muscle strength and function has been reported in older adults, with a threshold of 50 nmol/L appearing to be of musculoskeletal benefit [15]. Vitamin D deficiency ( < 25 nmol/L) is associated with an increased risk of sarcopenia [16], and positive correlations with skeletal muscle strength and function are generally observed in older adult populations. A systematic review with meta-analyses of 22 observational studies in older adults reported a slow walking speed and timed up and go performance in people with severe vitamin D deficiency, deficiency, and insufficiency compared to people with normal vitamin D status (defined as > 75 nmol/L) [17]. The results of vitamin D randomised control trials in older adults are mixed with some reporting improvements and others reporting no benefit, with a recent systematic review of intervention trials finding no benefit of supplementation on indices of sarcopenia, including timed up and go and handgrip strength [18]. The majority of the research included in these reviews is from predominantly white populations in the USA, Europe and Australia, or did not report ethnicity. There is considerable uncertainty with respect to associations between vitamin D and health in non-white populations and more research is needed in order to clarify recommended intakes for different ethnic groups [19, 20]. According to the 2021 Census 9.3% of the population living in England and Wales identify as from an Asian ethnic group [21] however few studies have considered vitamin D status and associated health in Asian communities living in the UK and the relationship between muscle strength, function and vitamin D status in South Asian women is unknown.

This study tests the hypothesis that there is a positive association between vitamin D status and muscle strength and physical function in South Asian women (Indian and Pakistani) aged ≥ 60 years living in the UK.

## Methods

### Study design

This cross-sectional study was designed to investigate the relationship between vitamin D status, muscle strength and physical function in UK South Asian women aged ≥ 60 years. This cross-sectional study was conducted between January 2018 and May 2018 and used convenience sampling. Ethical approval was gained from the University of Sheffield’s Medical School Research Ethics Committee (reference number 015586).

### Participants and recruitment

The study recruited community-dwelling women. The inclusion criteria were female, of South Asian background (Indian and Pakistani), aged ≥ 60 years, and able to give informed written and verbal consent. Given the cross-sectional nature of the study women were eligible for inclusion irrespective of their nutritional supplement use. Women were excluded if they were unable to communicate, were severely visually impaired/blind (for safety reasons), were unable to walk or were institutionalized. Participants were given the opportunity to discuss the study with their families before giving consent. All measures were undertaken by one researcher (SZ).

The recruitment was undertaken in two locations in the North of England: Sheffield and Greater Manchester (approximate Latitude 53.4°N), both of which have large South Asian communities. Convenience sampling was used to recruit the potential participants and recruitment was achieved through a variety of methods. Leaflets advertising the study were prepared in English and Urdu language and distributed around the South Asian community. In person recruitment sessions were held at South Asian community centres, Indian temples (Gurdwara) and mosques, using the preferred language of the community group and potential participants were given a participant information sheet. Interested and eligible participants were invited to meet with the researcher on a single appointment visit at their preferred location (participant home, community centre, temple or/and mosque) where informed written consent was obtained prior to the study assessments. On completion of the study participants were invited to enter a prize draw containing five prizes of £100, £75, £50, £25, £25 high street vouchers. Due to the convenience sampling nature of the study no formal power calculation was undertaken but a recruitment target of 120 participants was set pragmatically.

### Anthropometry

Information around participant demographics, anthropometrics, presence of comorbidities and the use of supplementation were collected during the face-to-face interview. Height was measured in centimetres using a portable stadiometer (Seca 213 Leicester portable height measure, UK) and body mass was measured in kilograms using a Tanita weighing scale (Tanita, BC-601). Waist and mid-upper arm circumference was measured in centimetres using a plastic measuring tape. The classification of World Health Organisation was used to define body mass index (BMI) thresholds (kg/m^2^) [22].

### Muscle strength and function assessment

Handgrip strength (kg) was measured using a Jamar handheld dynamometer (Jamar, Lafayette Instrument Company, La Fayette, IL, USA). Three consecutive readings were taken in a standing position using the right hand hanging free on the side of body with a one-minute interval between each attempt [23]. The maximum grip strength of the three attempts was used for analysis [24].

The single chair stand test (s) was used to examine lower limb muscle strength. The time to stand up from a straight back chair with arms folded around the chest and sit back down was measured. The repeated chair stand test was the time taken to perform single chair stand test five times consecutively [25].

The timed up and go test (TUG) was used to examine mobility, muscle function and walking speed [26]. This was the time taken to stand up from a chair, walk 3 m straight, turn around a marker, walk back to the chair and sit down.

The balance test assesses functional capacity of the lower limbs. It consists of side by side, semi-tandem and tandem test [25]. The side by side test evaluates a persons ability to maintain balance in a standing position with feet together for 10 s. Participants scored 1 if held the position for 10 s, otherwise participants scored 0 if they were unable to hold the position for 10 s or did not attempt the balance test. The semi-tandem test involves standing with the side of the heel of one foot touching the big toe of other foot for 10 s and scored the same as the side by side test. Tandem test involves standing with heel of one foot in front of and touching the toes of other foot for about 10 s. For the tandem test the participant scored 2 if balance was held for 10 s, scored 1 if held for 3–9.99 s and 0 if held for less than 3 s or not attempted. The total balance test score is the sum of all these three tests.

### Diet intake analyses

A 24-h single diet recall using the multiple pass method was used to assess dietary intake [27]. Participants were asked to recall all foods, beverages and snacks consumed in the previous 24 h. Participants were given about five minutes to think and recall their diet prior to the start of the interview which was conducted in their preferred language. Portion size and food type was identified and noted in a code form using a food record sheet. Data were analysed using DietPlan7 (Forestfield Software Ltd, UK). However the results are not presented as an analysis of 37 randomly selected food recalls revealed significant under-reporting of energy intake by the majority of participants, as determined using the Harris Benedict equation and cut-offs described by Black [28]. The food recalls were therefore deemed unreliable.

### Blood 25(OH) D measurement

A fingerprick blood spot was collected from each participant using a vitamin D blood spot kit [29]. Dried blood spots were sent directly for analysis by a service provider (Department of Clinical Biochemistry, Sandwell and West Birmingham Hospital, UK). The blood 25(OH)D concentration was measured as total 25-hydroxyvitamin D (both 25(OH)D_2_ and 25(OH)D_3_) analysed using liquid chromatography tandem mass spectrometry, using a method previously reported and validated [30–32]. The Institute of Medicine classification was used to define vitamin D inadequacy/deficiency (< 50 nmol/L) and sufficiency (≥ 50 nmol/L) [15].

### Statistical analysis

With a convenience sample of 120 participants, we would be able to detect a moderate effect size of 0.5 with approximately 80% power between handgrip strength, single chair stands, repeated chair stands, and timed up and go, and vitamin D status (no adjustments were made for multiple testing).

All analyses were carried out using Stata version 18. Statistical significance was set at the 5% level. To account for multiple testing amongst the five outcome measures, Holm-Bonferroni adjustment was used to control the family-wise error rate. This involved a step-down procedure testing vitamin D status parameter estimates for each outcome, such that the* p*-values were placed in ascending order, and sequentially tested against adjusted significance levels at each step until non-significance was reached. Holm-Bonferroni adjusted* p*-values were presented alongside the unadjusted* p*-values in the regression results.

Analysis models included complete cases only. Multiple imputation (MI) analysis, using chained equations, to impute missing data (single chair stand, repeated chair stand and balance test) was performed as a sensitivity analysis. For the MI analysis, m = 20 imputed datasets were created using an imputation model containing outcome variables, model covariates and auxiliary variables presented in Table 1 (it is suggested by White et al. [33] that the number of imputed data sets should be at least as large as the percentage of subjects with any missing data, therefore as 15% of participants had any missing data, m = 20 imputed datasets was selected). The models detailed in the main analysis were conducted for each imputed dataset with the results combined using Rubin’s rules [34]. The full MI analysis results are presented in the supplementary material (Table 5).

## Results

### Participants’ characteristics

**Fig. 1:**
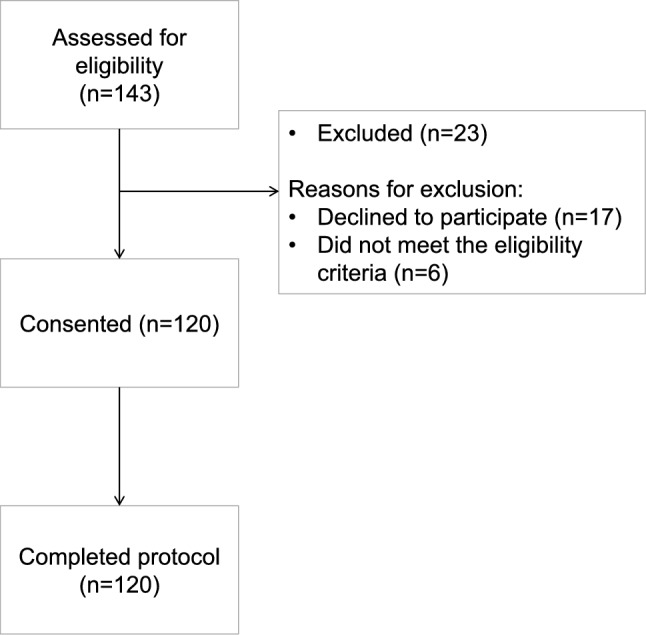
CONSORT flow-diagram showing participant recruitment

In total, one hundred and twenty women were successfully recruited to the study (Fig. 1). One-hundred and forty-three women were initially screened for inclusion, 6 of whom did not meet the eligibility criteria and 17 declined to participate after being fully informed about the study. Table 1 summarizes the characteristics of all the enrolled participants, including when split by vitamin D status. The median blood 25(OH)D concentration of the women was 53.0 nmol/L (data not shown). Forty-seven percent (n = 56) of the women had inadequate/deficient vitamin D status (< 50 nmol/L). The median age (IQR) of the women at the time of recruitment was 66 years (64–73). Geographically, there were higher rates of vitamin D inadequacy/deficiency in participants from Greater Manchester compared with Sheffield. The proportion of Indian and Pakistani women in the two vitamin D status categories were similar (Table 1). BMI, waist circumference and mid upper arm circumference of the women was similar according to vitamin D status. Ninety percent of women were overweight or obese, none were underweight. Fifty-two percent of the women reported no history of formal education. The median number of comorbidities reported was two and there was no difference in the total number of comorbidities according to vitamin D status. The most commonly self-reported morbidities were arthritis (70%), hypertension (59.2%), and diabetes (50.8%).

Forty-nine women (40.8%) reported regular consumption of vitamin D supplements of whom 84% had sufficient 25(OH)D concentration and 16% had inadequate/deficient status. Twenty-four % of women from Greater Manchester and 85% of women from Sheffield reported consumption of vitamin D supplements. In those women who reported that they did not consume vitamin D supplements 32% had a sufficient vitamin D status and 68% percent had inadequate/deficient vitamin D status. In women who consumed a vitamin D supplement the median blood 25(OH)D concentration was higher than in the non-consumers (83.1 nmol/L vs 30.8 nmol/L respectively) (data not shown).

### Muscle strength and function according to vitamin D status

Muscle strength and functional performance was assessed in the population as a whole and according to the vitamin thresholds of 25-hydroxyvitamin D < or ≥ 50 nmol/L (Table 2). The missing data for some tests (single chair stand test = 8 women; repeated chair stand test = 16 women and balance test = 2 women) was due to women being unable or unwilling to perform these tests. The participants who had vitamin D sufficient status on average completed the single chair stand, repeated chair stand, and timed up and go test faster than those who had inadequate status (Table 2). A greater proportion of participants who had vitamin D sufficient status scored a 4 (the maximum) on the balance test compared to participants with deficient vitamin D status. In contrast, there was little difference in the capacity of handgrip strength (kg) between participants with inadequate/deficient and sufficient levels.

Participant characteristics and outcome measures were summarised using descriptive statistics, displayed in Tables 1 and 2 respectively. Continuous data were reported as the number and percentage of complete records, mean, standard deviation, median, interquartile range, minimum and maximum, while categorical data were reported as counts and percentages. These were summarised by vitamin D sufficiency and overall.

**Table 1:** Participant characteristics in the total population and according to vitamin D status

Characteristic	Inadequate/deficient (< 50 nmol/L) (n = 56)	Sufficient (≥ 50 nmol/L) (n = 64)	Total (n = 120)
*Age (Years)*			
N (% data available)	56 (100.0)	64 (100.0)	120 (100.0)
Mean (SD)	68.0 (6.6)	69.5 (7.7)	68.8 (7.2)
Median (IQR)	66 (64–70)	66 (64–75)	66 (64–73)
Min, Max	60, 87	60, 87	60, 87
*Ethnicity, N (%)*			
N (% data available)	56 (100.0)	64 (100.0)	120 (100.0)
Indian	17 (30.4)	23 (35.9)	40 (33.3)
Pakistani	39 (69.6)	41 (64.1)	80 (66.7)
*Locality, N (%)*			
N (% data available)	56 (100.0)	64 (100.0)	120 (100.0)
Sheffield	7 (12.5)	26 (40.6)	33 (27.5)
Greater Manchester	49 (87.5)	38 (59.4)	87 (72.5)
*Waist circumference (cm)*			
N (% data available)	56 (100.0)	64 (100.0)	120 (100.0)
Mean (SD)	108.6 (12.7)	106.2 (10.1)	107.4 (11.4)
Median (IQR)	108 (100–120)	106 (100–112)	108 (100–114)
Min, Max	73, 137	77, 137	73, 137
*Mid upper arm circumference (cm)*			
N (% data available)	56 (100.0)	64 (100.0)	120 (100.0)
Mean (SD)	33.1 (4.5)	31.8 (5.3)	32.4 (5.0)
Median (IQR)	32 (30–37)	32 (29–35)	32 (29–36)
Min, Max	24, 42	12, 45	12, 45
*BMI (kg/m*^*2*^*)*			
N (% data available)	56 (100.0)	64 (100.0)	120 (100.0)
Mean (SD)	32.8 (6.0)	31.6 (5.4)	32.2 (5.7)
Median (IQR)	33 (28–37)	32 (28–35)	32 (28–36)
Min, Max	21, 46	22, 47	21, 47
*BMI (WHO categories), N (%)*			
Underweight (< 18.5)	0 (0.0)	0 (0.0)	0 (0.0)
Healthy (18.5–24.9)	7 (12.5)	5 (7.8)	12 (10.0)
Overweight (25–29.9)	13 (23.2)	21 (32.8)	34 (28.3)
Obese (> = 30)	36 (64.3)	38 (59.4)	74 (61.7)
*Education, N (%)*			
N (% data available)	56 (100.0)	64 (100.0)	120 (100.0)
None	35 (62.5)	28 (43.8)	63 (52.5)
Primary	15 (26.8)	23 (35.9)	38 (31.7)
Secondary	5 (8.9)	9 (14.1)	14 (11.7)
University	1 (1.8)	4 (6.2)	5 (4.2)
*Vitamin D supplement use, N (%)*			
N (% data available)	56 (100.0)	64 (100.0)	120 (100.0)
Consumer	8 (14.3)	41 (64.1)	49 (40.8)
Non-consumer	48 (85.7)	23 (35.9)	71 (59.2)
*No. of comorbidities per participant*			
N (% data available)	56 (100.0)	64 (100.0)	120 (100.0)
Mean (SD)	2.3 (1.3)	2.3 (1.1)	2.3 (1.2)
Median (IQR)	2 (1–3)	2 (2–3)	2 (1–3)
Min, Max	0, 5	0, 6	0, 6
*Comorbidities (Not mutually exclusive), N (%)*			
Arthritis, N (%)	40 (71.4)	44 (68.8)	84 (70.0)
Hypertension, N (%)	32 (57.1)	39 (60.9)	71 (59.2)
Diabetes, N (%)	30 (53.6)	31 (48.4)	61 (50.8)
Asthma, N (%)	11 (19.6)	16 (25.0)	27 (22.5)
Heart disease, N (%)	9 (16.1)	8 (12.5)	17 (14.2)
Liver disease, N (%)	0 (0.0)	0 (0.0)	0 (0.0)
Kidney disease, N (%)	2 (3.6)	3 (4.7)	5 (4.2)
Cancer, N (%)	7 (12.5)	5 (7.8)	12 (10.0)

“n” represents the number, kg/m^2^ is kilogram/meter square, BMI = body mass index

The age of participants is the age in years at the time of recruitment

**Table 2:** Muscle strength and function tests in the total population and according to vitamin D status

Outcome variable	Inadequate/deficient (< 50 nmol/L)	Sufficient (≥ 50 nmol/L)	Total (n = 120)
	(n = 56)	(n = 64)	
*Handgrip strength (kg)*			
N (% data available)	56 (100.0)	64 (100.0)	120 (100.0)
Mean (SD)	17.9 (4.6)	18.6 (4.7)	18.2 (4.7)
Median (IQR)	18.0 (15.0 – 20.3)	18.8 (15.4 – 21.1)	18.1 (15.2 – 20.9)
Min, Max	6.9, 31.2	5.0, 32.6	5.0, 32.6
*Normalised handgrip strength (kg/height*^*2*^*)*			
N (% data available)	56 (100.0)	64 (100.0)	120 (100.0)
Mean (SD)	7.6 (1.9)	7.9 (1.9)	7.8 (1.9)
Median (IQR)	7.7 (6.5 – 8.7)	7.8 (6.9 – 9.0)	7.7 (6.8 – 8.9)
Min, Max	3.6, 11.4	2.2, 13.1	2.2, 13.1
*Single chair stand (s)*			
N (% data available)	52 (92.9)	60 (93.8)	112 (93.3)
Mean (SD)	5.0 (2.0)	4.2 (1.9)	4.6 (2.0)
Median (IQR)	4.7 (3.6 – 5.6)	3.7 (3.1 – 4.9)	4.0 (3.3 – 5.3)
Min, Max	2.2, 11.7	1.8, 10.4	1.8, 11.7
*Repeated chair stand test (s)*			
N (% data available)	48 (85.7)	56 (87.5)	104 (86.7)
Mean (SD)	25.2 (8.3)	21.2 (7.8)	23.0 (8.2)
Median (IQR)	23.2 (20.2 – 28.1)	19.8 (15.6 – 24.3)	21.8 (18.1 – 26.0)
Min, Max	12.2, 62.2	11.1, 51.6	11.1, 62.2
*Timed up and go test (s)*			
N (% data available)	56 (100.0)	64 (100.0)	120 (100.0)
Mean (SD)	18.3 (9.3)	16.5 (11.1)	17.3 (10.3)
Median (IQR)	15.1 (12.6 – 20.5)	13.1 (10.5 – 16.6)	14.3 (11.7 – 19.4)
Min, Max	4.8, 52.1	6.9, 78.3	4.8, 78.3
*Balance test score, N (%)*			
Data available (%)	55 (98.2)	63 (98.4)	118 (98.3)
1	7 (12.7)	4 (6.4)	11 (9.3)
2	13 (23.6)	13 (20.6)	26 (22.0)
3	16 (29.1)	11 (17.5)	27 (22.9)
4	19 (34.6)	35 (55.6)	54 (45.8)

Eight participants were unable or unwilling to perform the single chair stand test

Sixteen participants were unable or unwilling to perform the repeated chair stand

Two participants were unable or unwilling to perform the balance test

### Relationship between vitamin D status, muscle strength and function in overall participants

The relationship between vitamin D status and muscle strength and function in the population was examined using regression, correcting for age, BMI, diabetes and presence of arthritis as shown in Table 3. There was no between group evidence of difference in handgrip strength or timed up and go test performance between participants with sufficient or inadequate/deficient status. Those sufficient in vitamin D demonstrated a 0.79 (95% CI − 0.93–2.51) kg increase in grip strength, although this result does not show a statistically significant difference between the groups. Participants with sufficient vitamin D status took less time to perform single chair stand (*p* = 0.015; adjusted *p* = 0.045), repeated chair stand (*p* = 0.002; adjusted *p* = 0.01) and balance test (*p* = 0.010; adjusted *p* = 0.04) compared to those with inadequate/deficient status. Returning to the original scale, on average those sufficient with vitamin D had a decrease in time for single chair stands by − 0.82 (95% CI − 1.49 to − 0.16) seconds, decrease in time for repeated chair stand by − 4.46 (95% CI − 7.31 to − 1.61) seconds, and decrease in time for the timed up and go test by − 2.28 (95% CI − 5.09–0.54) seconds. The results of the single chair and repeated chair stand test suggest a significant difference in times when comparing vitamin D groups. Participants with sufficient status were 2.54 (95% CI 1.25–5.17) times more likely to perform balance test better than those with inadequate/deficient status.

To determine associations between vitamin D status and muscle strength and function, multivariable linear regression models were conducted for continuous outcomes, adjusting for the following independent variables: age, BMI, arthritis and diabetes. Non-standardised regression coefficients are presented in Table 3. Unadjusted linear regression models were also conducted and are presented in the supplementary material. The assumptions of normality and homoscedasticity of residuals of linear regression models were checked using graphical methods. The residuals of the regression models involving single chair stands, repeated chair stands, and timed up and go outcomes were non-normally distributed. The data for these outcomes were positively skewed and so, were transformed using a log transformation prior to fitting the regression models. Following this, the residuals of all models satisfied the normality and homoscedasticity assumptions. The results of the linear regression models were presented in the transformed scale (where applicable), and marginal effects of vitamin D status on the original scale were calculated with 95% confidence intervals. For the balance test, as this is scored on a discrete scale, ordinal regression was used, adjusting for the same variables as stated above. The assumption of proportional odds was checked using the Brant test. The Brant test provided non-significant results (*p*-value > 0.05) implying that the proportional odds assumption was satisfied.

**Table 3:** Association between vitamin D status and muscle strength and function

Outcome variable	Adjusted regression coefficients (95% CI)	*p*-value (Holm-Bonferroni adjusted* p*-value)
*Handgrip strength (kg)*		
Vitamin D status		
Deficient (< 50 nmol/L)	Reference	
Sufficient (> = 50 nmol/L)	0.79 (− 0.93–2.51)	0.365 (0.365)
Age (years)	− 0.06 (− 0.18–0.06)	0.305
BMI (kg/m^2^)	0.03 (− 0.13–0.18)	0.732
Arthritis		
Not Present	Reference	
Present	− 1.58 (− 3.50–0.33)	0.105
Diabetes		
Not Present	Reference	
Present	− 0.01 (− 1.77–1.75)	0.991
Single chair stand (log(s))*		
Vitamin D Status		
Deficient (< 50 nmol/L)	Reference	
Sufficient (> = 50 nmol/L)	− 0.179 (− 0.323 – − 0.035)	**0.015 (0.045)**
Age (years)	0.003 (− 0.008–0.013)	0.634
BMI (kg/m^2^)	0.007 (− 0.005–0.020)	0.245
Arthritis		
Not Present	Reference	
Present	0.081 (− 0.077–0.239)	0.311
Diabetes		
Not Present	Reference	
Present	− 0.069 (− 0.218–0.079)	0.357
Repeated chair stands (log(s))*		
Vitamin D Status		
Deficient (< 50 nmol/L)	Reference	
Sufficient (> = 50 nmol/L)	− 0.192 (− 0.315 – − 0.070)	**0.002 (0.01)**
Age (years)	0.005 (− 0.004–0.014)	0.304
BMI (kg/m^2^)	0.001 (− 0.009–0.012)	0.834
Arthritis		
Not Present	Reference	
Present	0.051 (− 0.082–0.184)	0.450
Diabetes		
Not Present	Reference	
Present	− 0.064 (− 0.191–0.062)	0.315
Timed up and go (log(s))*		
Vitamin D Status		
Deficient (< 50 nmol/L)	Reference	
Sufficient (> = 50 nmol/L)	− 0.124 (− 0.282–0.034)	0.122 (0.244)
Age (years)	0.015 (0.004–0.026)	0.010
BMI (kg/m^2^)	0.018 (0.004–0.032)	0.014
Arthritis		
Not Present	Reference	
Present	0.117 (− 0.059–0.293)	0.190
Diabetes		
Not Present	Reference	
Present	0.122 (− 0.041–0.284)	0.140
Balance test score**		
Vitamin D Status		
Deficient (< 50 nmol/L)	Reference	
Sufficient (> = 50 nmol/L)	2.54 (1.25–5.17)	**0.010 (0.04)**
Age (years)	0.91 (0.86–0.96)	< 0.001
BMI (kg/m^2^)	0.98 (0.92–1.04)	0.424
Arthritis		
Not Present	Reference	
Present	0.87 (0.41–1.85)	0.734
Diabetes		
Not Present	Reference	
Present	1.01 (0.49–2.06)	0.980

*Outcome variable was transformed to meet normality assumptions of linear regression

**Ordinal regression used—results presented as odds ratio

The MI analysis provided slightly differing results for the single chair stand, repeated chair stand and balance test (Table 5, Supplementary material). With imputation for missing values participants with sufficient vitamin D status still took less time to complete the single chair stand (− 0.73 [95% CI − 1.40 to − 0.06; *p* = 0.033] seconds) and repeated chair stand (− 3.84 [95% CI − 7.19 to − 0.48; *p* = 0.025] seconds), however the MI analysis demonstrated non-significant results after the Holm-Bonferroni procedure (adjusted *p*-values: *p* = 0.104 and *p* = 0.102 respectively). Participants sufficient in vitamin D status were 2.58 (95% CI 1.27–5.25) times more likely to perform better on the balance test. The MI results for the balance test remained statistically significant (*p* = 0.009; adjusted *p* = 0.045). The results were unchanged for participant handgrip strength and timed up and go, due to no missing data observed for these outcomes.

## Discussion

This study describes the relationships between vitamin D status and muscle strength and function in South Asian women aged ≥ 60 years living in the North of England; to our knowledge this is the largest sample of this population directly assessed for this relationship. The main findings of this cross-sectional observational study are (i) associations between vitamin D status and some aspects of lower limb muscle strength, but not with handgrip strength and timed up and go, and (ii) a high prevalence of vitamin D deficiency/inadequacy in unsupplemented women.

This study observed that South Asian women aged ≥ 60 years with sufficient vitamin D status (≥ 50 nmol/L) performed better on the balance and the single and repeated chair stand tests than women with inadequate/deficient vitamin D status. Participants with sufficient vitamin D status were 2.54 times more likely to perform better on the balance test compared to women with an inadequate/deficient vitamin D status. These observations are in agreement with several other groups. Akdeniz et al. [35] made similar observations in a population of women aged ≥ 60 years living in Turkey, whereby women with serum 25(OH)D of 50 nmol/L and above displayed significantly better balance than those with lower levels of vitamin D. In a longitudinal study of geriatric outpatients in Turkey poorer balance was observed in those with severe vitamin D deficiency and improvements in balance were seen with improvement in vitamin D status [36]. In the current study in terms of lower limb muscle strength, participants with sufficient vitamin D status performed better on single and repeated chair stands than those with inadequate/deficient status. Others have similarly reported better lower limb muscle strength at 25(OH)D concentration ≥ 75 nmol/L in community dwelling women aged 60 years and above [24, 37–39]. The positive associations we have observed do not demonstrate causality, however balance and lower limb strength are important elements of falls prevention and there is evidence that supplemental vitamin D improves muscle strength, and balance [40] and reduces the risk of falls in older adults with low vitamin D status [41]. In contrast more recent large RCTs have found no benefit and the possibility of a negative effect of vitamin D supplementation on physical function in older adults [42, 43]. Questions have been raised with respect to the mode of administration, dose of vitamin D and optimal vitamin D status with respect to muscle strength, function and fracture risk [44]. There is some evidence that high dose vitamin D in already replete individuals and bolus doses of vitamin D are detrimental, and as with many micronutrients, there is suggestion of the existence of a U shaped relationship with respect to optimum status for health [45, 46]. Our findings are in line with IOM recommendations of vitamin D repletion for good musculoskeletal health [15].

In contrast to other studies we did not find an association with vitamin D status and TUG. Boye et al., [47] reported better TUG performance in older women (mean age 76.5 years) who attended emergency departments in hospitals in the Netherlands due to a fall and had higher serum 25(OH)D level (> 75 nmol/L) compared to women with serum 25(OH)D level < 25 nmol/L. Okuno and colleagues made similar observations in Japan in community-dwelling frail women aged 65 years and above with history of falls and reported better TUG performance at circulating 25(OH)D concentration ˃ 67.5 nmol/L [48]. Chuang et al. (2016) also reported an association between TUG and circulating 25(OH)D concentration in community dwelling Taiwanese women, and reported women with blood 25(OH)D concentration of 75 nmol/L performed better on the TUG test compared to those with a 25-OHD concentration of 30–50 nmol/L [24].

The present study did not detect any association between handgrip strength and vitamin D status. Those sufficient in vitamin D demonstrated a 0.79 (95% CI − 0.93–2.51) kg increase in grip strength, although this result does not show a statistically significant difference between the groups. Our observations should be interpreted with caution since the prevalence of arthritis was high in our population (70%) and this may have negatively impacted on the participants’ handgrip strength (− 1.58 (95% CI − 3.50–0.33) kg; *p* = 0.11). In addition, fifty percent of participants reported to have diabetes that might have impacted some indices of muscle strength and function caused by diabetes related neuropathy affects on skeletal muscle [49]. Nonetheless these results are in agreement with a number of other studies which have also reported lack of association between 25(OH)D concentration and handgrip strength in community-dwelling women aged 60 years and above [24, 37, 47, 50]. It is important to consider the current study within the context of the revised version of European Working Group on Sarcopenia in Older People 2 (EWGSOP2) which has reported the mean (SD) normative value of handgrip strength of 26.5(6.2) kg in women aged 60 years old [51]. Given the median age of our study population the median handgrip strength was lower than expected, which may be indicative of sarcopenia, which coupled with the high prevalence of overweight and obesity raises the possibility of sarcopenic obesity in this population which warrants further investigation.

The current study confirms previous reports of hypovitaminosis D in UK South Asian post-menopausal women [7, 52] and as with other studies this was particularly evident in those not consuming vitamin D supplements [14]. A smaller proportion of women from Greater Manchester were using vitamin D supplements compared to women recruited in Sheffield. Our convenience recruitment strategy is prone to selection bias, nonetheless a regional variation in GP prescribing of vitamin D supplements has been reported by others [53] and may be indicative of inequity in prescribing practices. Similar findings were seen in the UK Biobank data which has reported regional differences in the vitamin D supplement usage in UK South Asians [8].

The main limitation in the design of the current study is that causality and temporality cannot be inferred from cross-sectional studies. Longitudinal studies and randomised controlled trials would help understand the impact of confounding factors on the observed findings and could help establish cause and effect. Other limitations include the lack of dietary intake data and no measure of sunlight exposure. Dietary intake was significantly under-reported by the women included in our study to the extent that the majority of records were unusable and therefore we were unable to correct, not only for dietary vitamin D intake itself, but also for dietary intake of other nutrients known to be important for musculoskeletal health such as protein and calcium. This highlights the difficulty in collecting accurate estimates of intake and the need for validated culturally sensitive dietary assessment tools [54]. Whilst sunlight exposure was not measured, all women wore culturally appropriate dress that limits skin exposure and assessments were made in the winter months when sunlight exposure in this region is insufficient for cutaneous synthesis of vitamin D. Further limitations are that supplement use was self-reported which may introduce recall bias and social desirability bias; post-menopausal status was assumed given the age of the women and the use of hormone replacement therapy and other medications, that may have impacted the findings, was not collected. Nonetheless those with better vitamin D status had better lower limb strength which supports a rationale for vitamin D supplementation in populations at particular risk of vitamin D deficiency. No formal power calculation was performed for this study, and this may limit the detection of associations. We are not reporting retrospective power calculations as we are reporting on a convenience sample and researchers wishing to use the data to calculate sample size should be mindful of this.

A strength of the study is that we managed to recruit a larger sample size than is usually achieved in this under-researched population group. The participants included were all South Asian women aged ≥ 60 years which limits the generalisability of the results to other ages and populations; however this population was deliberately selected due the lack of research in the population. Inconsistencies with the existing literature may reflect the existence of differential associations in different ethnic groups, and our observations may help inform optimal vitamin D intakes and status for women of South Asian origin. Further strengths of the study are that participants were found to have a wide range of vitamin D status, and that we recruited different South Asian communities (Pakistani and Indian) at two geographical locations demonstrating the feasibility of research in these communities. Our research suggests that vitamin D supplementation is a valuable strategy for the maintenance of vitamin D status in this particular population at high risk of vitamin D deficiency. Future studies should investigate whether relationships we have observed are causal and whether supplementation can remedy impaired muscle strength and function in this group.

## Conclusions

- Favourable associations exist between vitamin D status and some aspects of lower limb muscle strength and function in South Asian women aged ≥ 60 years.
- There is a high prevalence of vitamin D deficiency in unsupplemented South Asian women aged ≥ 60 years.
- Vitamin D supplementation in this population appears to be an effective strategy for achieving vitamin D adequacy.

## Supplementary Information

Below is the link to the electronic supplementary material.

Supplementary file1 (DOCX 18 KB)

## Data Availability

Not applicable.
