# Supplementary material for: Vitamin D, muscle strength and function in South Asian women aged ≥ 60 years living in the North of England: a cross-sectional observational study
Source: Eur J Nutr. 2025 Oct 9;64(7):291. doi: 10.1007/s00394-025-03787-7 (PMC12511266; doi:10.1007/s00394-025-03787-7)
Supplement: Supplementary file 1 — Supplementary file1 (DOCX 18 KB) [file 394_2025_3787_MOESM1_ESM.docx]

**Supplementary Material**

**Table 4.** Association between vitamin D status and muscle strength and function (Unadjusted regression models)

| **Outcome Variable** | **Unadjusted regression coefficients (95% CI)** | **p-value** |
| --- | --- | --- |
| **Handgrip strength (kg)**  Vitamin D Status  Deficient (<50nmol/L)  Sufficient (>=50nmol/L) | Reference  0.71 (-0.99 – 2.40) | 0.412 |
| **Single chair stand (log(s))***  Vitamin D Status  Deficient (<50nmol/L)  Sufficient (>=50nmol/L) | Reference  -0.184 (-0.326 – -0.041) | 0.012 |
| **Repeated chair stands (log(s))***  Vitamin D Status  Deficient (<50nmol/L)  Sufficient (>=50nmol/L) | Reference  -0.180 (-0.300 – -0.060) | 0.004 |
| **Timed up and goes (log(s))***  Vitamin D Status  Deficient (<50nmol/L)  Sufficient (>=50nmol/L) | Reference  -0.133 (-0.297 – 0.032) | 0.112 |
| **Balance test score****  Vitamin D Status  Deficient (<50nmol/L)  Sufficient (>=50nmol/L) | Reference  2.03 (1.03 – 3.99) | 0.040 |
| *Outcome variable was transformed to meet normality assumptions of linear regression  **Ordinal regression used – results presented as odds ratio | | |

Multiple Imputation analysis - Regression coefficients are presented in Table 5 alongside the complete-case analysis for comparison.

**Table 5:** Association between vitamin D status and muscle strength and function; multiple imputation results compared with complete case analysis.

| **Outcome Variable** | **Complete-case analysis** | | **Multiple imputation analysis** | |
| --- | --- | --- | --- | --- |
|  | **Adjusted regression coefficients (95% CI)** | **p-value (Holm-Bonferroni adjusted p-value)** | **Adjusted regression coefficients (95% CI)** | **p-value (Holm-Bonferroni adjusted p-value)** |
| **Handgrip strength (kg)**  Vitamin D Status  Deficient (<50nmol/L)  Sufficient (>=50nmol/L)  Age (years)  BMI (kg/m^2)  Arthritis  Not Present  Present  Diabetes  Not Present  Present | Reference  0.79 (-0.93 – 2.51)  -0.06 (-0.18 – 0.06)  0.03 (-0.13 – 0.18)  Reference  -1.58 (-3.50 – 0.33)  Reference  -0.01 (-1.77 – 1.75) | 0.365 (0.365)  0.305  0.732  0.105  0.991 | Reference  0.79 (-0.93 – 2.51)  -0.06 (-0.18 – 0.06)  0.03 (-0.13 – 0.18)  Reference  -1.58 (-3.50 – 0.33)  Reference  -0.01 (-1.77 – 1.75) | 0.365 (0.365)  0.305  0.732  0.105  0.991 |
| **Single chair stand (log(s))***  Vitamin D Status  Deficient (<50nmol/L)  Sufficient (>=50nmol/L)  Age (years)  BMI (kg/m^2)  Arthritis  Not Present  Present  Diabetes  Not Present  Present | Reference  -0.179 (-0.323 – -0.035)  0.003 (-0.008 – 0.013)  0.007 (-0.005 – 0.020)  Reference  0.081 (-0.077 – 0.239)  Reference  -0.069 (-0.218 – 0.079) | **0.015 (0.045)**  0.634  0.245  0.311  0.357 | Reference  -0.164 (-0.315 – -0.013)  0.003 (-0.008 – 0.014)  0.011 (-0.003 – 0.024)  Reference  0.135 (-0.031 – 0.301)  Reference  -0.039 (-0.193 – 0.116) | 0.034 (0.102)  0.644  0.118  0.110  0.619 |
| **Repeated chair stands (log(s))***  Vitamin D Status  Deficient (<50nmol/L)  Sufficient (>=50nmol/L)  Age (years)  BMI (kg/m^2)  Arthritis  Not Present  Present  Diabetes  Not Present  Present | Reference  -0.192 (-0.315 – -0.070)  0.005 (-0.004 – 0.014)  0.001 (-0.009 – 0.012)  Reference  0.051 (-0.082 – 0.184)  Reference  -0.064 (-0.191 – 0.062) | **0.002 (0.010)**  0.304  0.834  0.450  0.315 | Reference  -0.161 (-0.302 – -0.020)  0.003 (-0.008 – 0.013)  0.003 (-0.009 – 0.015)  Reference  0.15 (-0.008 – 0.301)  Reference  -0.032 (-0.176 – 0.112) | 0.026 (0.104)  0.601  0.637  0.063  0.663 |
| **Timed up and goes (log(s))***  Vitamin D Status  Deficient (<50nmol/L)  Sufficient (>=50nmol/L)  Age (years)  BMI (kg/m^2)  Arthritis  Not Present  Present  Diabetes  Not Present  Present | Reference  -0.124 (-0.282 – 0.034)  0.015 (0.004 – 0.026)  0.018 (0.004 – 0.032)  Reference  0.117 (-0.059 – 0.293)  Reference  0.122 (-0.041 – 0.284) | 0.122 (0.244)  0.010  0.014  0.190  0.140 | Reference  -0.124 (-0.282 – 0.034)  0.015 (0.004 – 0.026)  0.018 (0.004 – 0.032)  Reference  0.117 (-0.059 – 0.293)  Reference  0.122 (-0.041 – 0.284) | 0.122 (0.244)  0.010  0.014  0.190  0.140 |
| **Balance test score****  Vitamin D Status  Deficient (<50nmol/L)  Sufficient (>=50nmol/L)  Age (years)  BMI (kg/m^2)  Arthritis  Not Present  Present  Diabetes  Not Present  Present | Reference  2.54 (1.25 – 5.17)  0.91 (0.86 – 0.96)  0.98 (0.92 – 1.04)  Reference  0.87 (0.41 – 1.85)  Reference  1.01 (0.49 – 2.06) | **0.010 (0.040)**  <0.001  0.424  0.734  0.980 | Reference  2.58 (1.27 – 5.25)  0.91 (0.86 – 0.96)  0.98 (0.92 – 1.04)  Reference  0.88 (0.41 – 1.90)  Reference  0.99 (0.49 – 2.03) | **0.009 (0.045)**  <0.001  0.443  0.747  0.988 |
| *Outcome variable was transformed to meet normality assumptions of linear regression  **Ordinal regression used – results presented as odds ratio | | | | |
